# The Impact of Sarcopenia in the Long-Term Survival of Patients following Complex Endovascular Aortic Surgery for Thoracoabdominal Aortic Aneurysms

**DOI:** 10.3390/diagnostics14070751

**Published:** 2024-04-01

**Authors:** Georgios Sachsamanis, Judith Stahl, Karin Pfister, Wilma Schierling, Thomas Betz, Simon Jage

**Affiliations:** 1 Department of Vascular and Endovascular Surgery, University Medical Center Regensburg, 93053 Regensburg, Germany; judith.stahl@ukr.de (J.S.); karin.pfister@ukr.de (K.P.); wilma.schierling@ukr.de (W.S.); thomas.betz@ukr.de (T.B.); 2 Department of Radiology, University Medical Center Regensburg, 93053 Regensburg, Germany; simon.jage@ukr.de

**Keywords:** sarcopenia, skeletal muscle mass, total psoas muscle mass, thoracoabdominal, aneurysm

## Abstract

Objectives: Image-based sarcopenia has been the subject of recent studies, hypothesized as a prognostic factor for patients with thoracoabdominal aortic aneurysms. Methods and Materials: We conducted a single-center retrospective analysis of patients who underwent complex endovascular repair for thoracoabdominal aortic aneurysms between 2008 and 2016. CT image assessment was performed and patients were classified as sarcopenic and non-sarcopenic using two stratification methods: skeletal mass index (SMI) and total psoas muscle index (TPMI). According to sex, each patient was defined as sarcopenic if their SMI or TPMI was in the lowest third of the study group. The primary endpoint was impact of sarcopenia on perioperative mortality and long-term survival. Secondary endpoints were perioperative complications. Results: From a total of 155 patients, 135 were eligible for study. Overall, in-hospital mortality was 5.9% (8/135). The 30-day, 1-year, 3-year and 5-year mortality was 10.4% (14/135), 20% (27/135), 28.1% (38/135) and 31.1% (42/135), respectively. There was no difference in the long-term mortality rates between sarcopenic and non-sarcopenic patients regardless of the stratification method used (*p* = 0.4 for SMI and *p* = 0.2 for TPMI). According to SMI, 30-day mortality of sarcopenic patients was significantly lower in comparison to non-sarcopenic patients (1/45, 2.2% vs. 13/90, 14.4%, *p* = 0.028). Based on the total psoas muscle index, sarcopenic patients were at higher risk for development of pulmonary complications in comparison to non-sarcopenic patients postoperatively (*p* = 0.03). Conclusion: Using SMI and TPMI, sarcopenia was not associated with reduced long-term survival in patients undergoing complex endovascular repair for thoracoabdominal aortic aneurysms.

## 1. Introduction

Sarcopenia is defined as the age-related loss of skeletal muscle and strength, leading to reduced physical performance and overall diminished quality of life [1]. It is a condition presented mainly in elderly people; its development, however, may begin in earlier stages of life [2].

Sarcopenia has been the subject of many studies, being recognized as a prognostic factor in both malignant and non-malignant diseases [3,4,5]. Most reports utilize an image-based stratification tool with measurement of either skeletal muscle area (SMA) or total psoas muscle area (TPMA) at the level of the third lumbar vertebrae in order to classify patients as sarcopenic and non-sarcopenic. Reduced muscle mass before surgery has been associated with increased mortality and morbidity risk following major surgical procedures [6,7]. Several strategies exist for addressing sarcopenia and enhancing skeletal muscle, including the use of nutritional supplements and exercise [8]. However, there is currently no clear consensus on the preoperative management of sarcopenia.

Sarcopenia’s relationship with long-term survival has been extensively investigated in patients with abdominal aortic aneurysm (AAA), with many authors hypothesizing a potential relation between decreased skeletal muscle and long-term survival. Nevertheless, the available data remain inconclusive [9,10,11]. In a retrospective study from 2018, Indrakusuma et al. reported no association between psoas muscle area and patients with an asymptomatic infrarenal abdominal aortic aneurysm [9]. Similarly, the results of Waduud et al. demonstrated that total psoas muscle area is not a suitable tool for predicting mortality after AAA interventions, suggesting testing of other measures of frailty such as gait speed and grip strength [10]. However, a study from Hale et al. suggested that the presence of sarcopenia in CT images can indeed be an important predictor of long-term mortality in patients undergoing endovascular repair for AAA [11]. The role of sarcopenia in patients with thoracoabdominal aortic aneurysms (TAAAs) is currently unknown, with only a few studies addressing the subject [12,13,14].

The aim of this report is to test if there is a potential association between survival and reduced muscle mass in patients with TAAA following elective complex endovascular repair.

## 2. Materials and Methods

### 2.1. Patient Cohort

We conducted a single-center retrospective analysis of all patients who underwent endovascular repair of aortic pathologies in our institution between January 2008 and December 2016. Patients included in the study underwent elective endovascular repair of TAAAs using either fenestrated or branched endografts or a combination of both. Patients who received management in an emergency setting were excluded. All patients had undergone a preoperative computer tomography angiography (CTA) examination within six months prior to the procedure. Patients with poor quality or reduced field of view (FOV) of the CTA images were also excluded from the study. Collected data included patient’s demographics and medical history. Perioperative outcomes and follow-up data were additionally collected and analyzed. The institution’s ethical committee has approved this study (registration number 23-32-42-104).

### 2.2. Assessment of Skeletal Muscle

**Figure 1:**
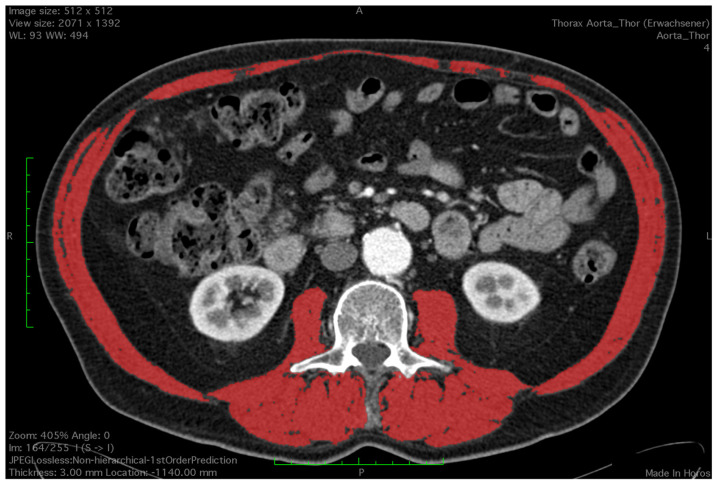
Measurement of skeletal muscle area using a semi-automated Houndsfield unit-based approach. Red colour depicts the measured area.

**Figure 2:**
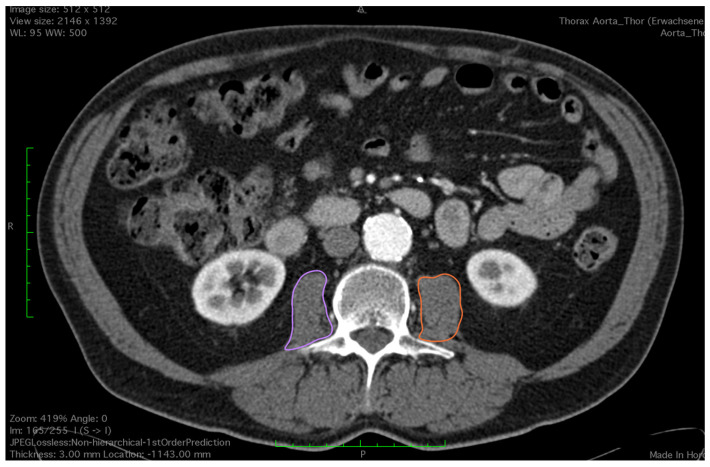
Outlining both of the psoas muscles for measurement of total psoas muscle area (purple and orange circles).

All CT images were analyzed by a trained observer, blind to the patients’ outcome. We used a semi-automated Hounsfield unit (HU)-based approach with manual correction. Skeletal muscle area (SMA) was measured at the level of the third lumbar vertebrae, which is an established landmark for measuring skeletal muscle mass in CT scans [15,16]. Both transversus processus had to be visible and two adjacent slices were analyzed. CT scans with or without contrast agent and ranging from 3–5 mm in slice thickness were analyzed using HorosTM (Version 3.3.6) and utilizing the ‘grow region’ tool. The HU threshold was set from −29 HU to +150 H for optimal muscle detection. Muscles at the level of L3 included the M. psoas, paraspinal, rectus abdominus and rectus oblique. Falsely selected areas such as visceral organs or subcutaneous tissue due to similar Hounsfield units were manually removed by the observer using the ‘brush tool’. The ‘brush tool’ allows manual editing of a region of interest (ROI). Additionally, total psoas muscle area (TPMA) was measured separately on the same two CT scan slices using the closed polygon tool. The outline of the ROI is marked with multiple manually added points which are then automatically joined into the desired shape. Right and left psoas muscle areas were measured separately (Figure 1 and Figure 2). Skeletal mass index (SMI) and total psoas mass index (TPMI) were obtained according to patient’s height (measured in meters) using the following formula: (SMA)/(patient’s height)^2^, and (TPMA)/(patient’s height)^2^. The impact of sarcopenia was assessed independently using SMI and TMPI values. Patients where first classified into two cohorts according to sex and each patient was defined as sarcopenic if their SMI or TPMI was in the lowest third of the study group. Males and females were then added to form a sarcopenic and a non-sarcopenic group for both SMI and TPMI values.

### 2.3. Study Endpoints

The primary endpoint of the study was perioperative mortality and mortality during follow-up. Patients were monitored at 30 days after discharge and then at 1, 3 and 5 years. Patients lost to follow-up were contacted via telephone or via the general practitioner during data acquisition. The secondary endpoints were postoperative major adverse events, such as cardiac failure, pulmonal complications and acute kidney injury.

### 2.4. Statistical Analysis

The analysis of the collected data was performed using SPSS for Windows (Version 27; SPSS Inc., Chicago, IL, USA). Continuous variables are presented as mean ± standard deviation (SD) and categorical variables as number and % percentage. Statistical analysis was undertaken using crosstabs, non-parametric statistics, Kaplan–Meier analysis and Cox regression analysis. Non-parametric data were compared using the Whitney U test, while categorical data were compared using the Pearson chi-square test and Fisher’s exact test as appropriate. Statistically analyzed data were considered significant when *p* < 0.05.

## 3. Results

### 3.1. Cohort Characteristics

**Figure 3:**
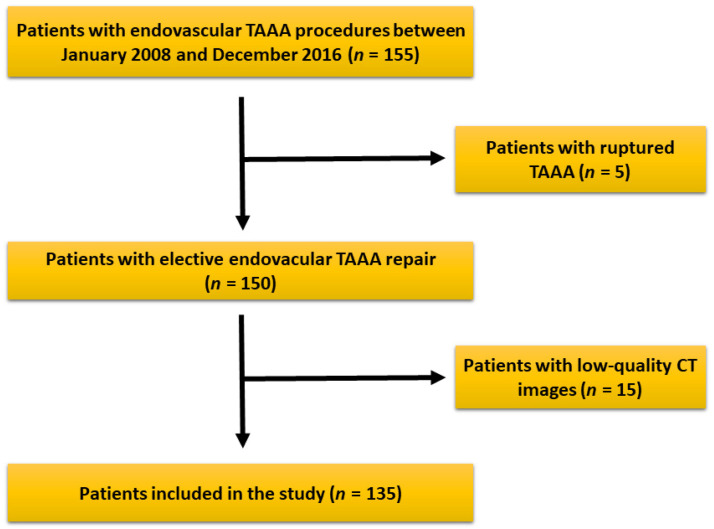
Patients’ selection criteria and eligibility for the study. After patient selection, a total of 135 patients were included in the study.

After reviewing our records, 155 patients underwent endovascular repair of TAAA in our institution during the study period. Five patients had an emergency procedure and were excluded. Fifteen more patients were excluded due to low-quality or reduced field of view (FOV) of the CT images during assessment, leaving a total of 135 patients eligible for study (Figure 3). After image evaluation, median SMI and TPMI were 44.7 ± 9 cm^2^/m^2^ and 5.3 ± 1.6 cm^2^/m^2^ for the whole cohort, respectively. The total range was 28.1–76.1 cm^2^/m^2^ for SMI and 2.08–11.3 cm^2^/m^2^ for TPMI. The interquartile range was 37.9–49.7 cm^2^/m^2^ and 4–6.1 cm^2^/m^2^ for SMI and TPMI, respectively. The cutoff values to determine sarcopenia according to SMI measurements were 43.9 cm^2^ for males and 35.2 cm^2^ for females. Regarding TPMI, the cutoff values were 5.1 cm^2^ and 3.6 cm^2^ for males and females, respectively. Patient overall demographics and cohort characteristics according to SMI and TPMI are shown in Table 1 and Table 2, respectively. There was no statistical difference between male to female ratio, age and body mass index in sarcopenic and non-sarcopenic groups regardless of the measurement method used. A statistically significant association between type II TAAA and reduced muscle mass was demonstrated after patient stratification with both SMI (*p* = 0.01) and TPMI (*p* < 0.01) measurement methods.

### 3.2. Primary Endpoints

**Figure 4:**
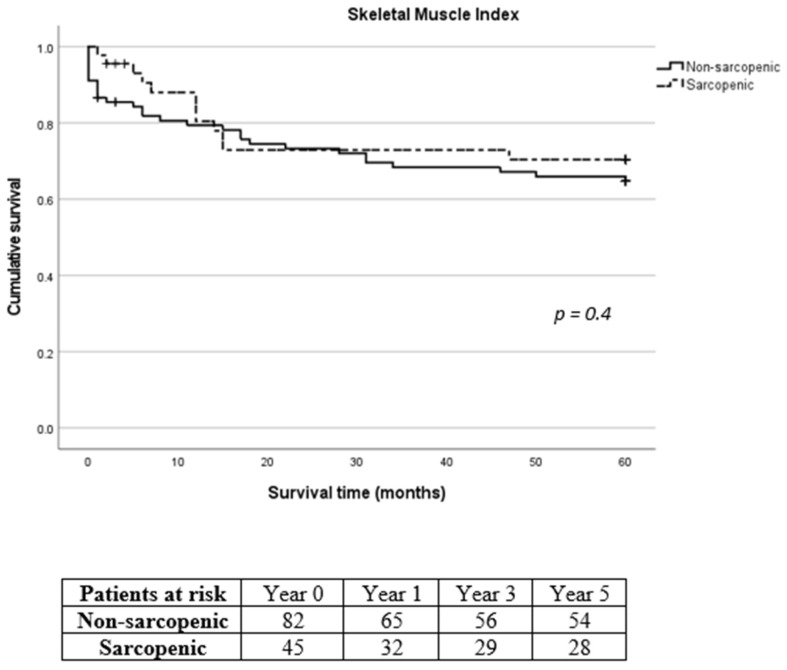
Long-term survival of sarcopenic and non-sarcopenic patients after stratification using skeletal muscle index. The Kaplan–Meier curves were compared using the log-rank test.

**Figure 5:**
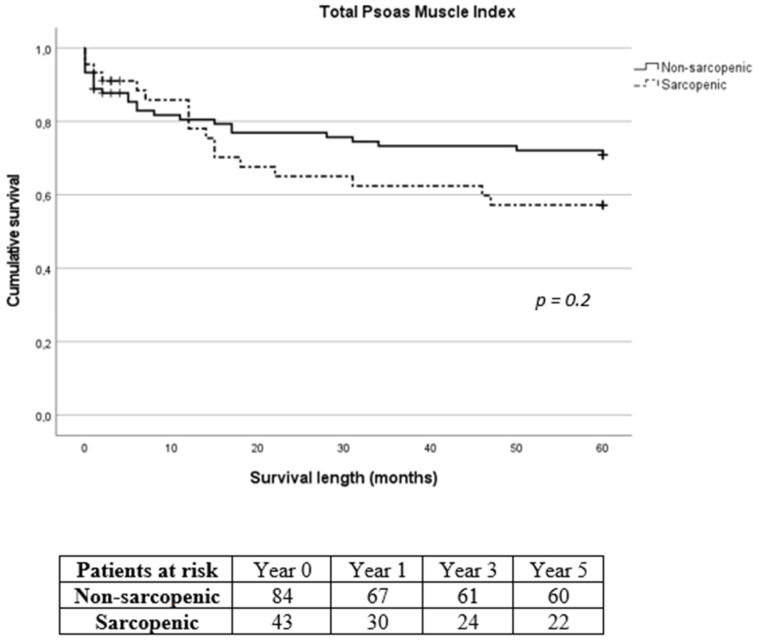
Long-term survival of sarcopenic and non-sarcopenic patients after stratification using total psoas muscle index. The Kaplan–Meier curves were compared using the log-rank test.

Mean intensive care unit (ICU) and hospital length of stay following SMI stratification were 6.1 ± 8.4 days and 21.9 ± 15 days for the non-sarcopenic group and 5.6 ± 4.4 days and 24.5 ± 8.9 days for the sarcopenic group, respectively (*p* = 0.016 for hospital length of stay). According to TPMI, ICU and hospital length of stay were 5.9 ± 8.3 days and 21.9 ± 13.1 days for the non-sarcopenic group and 6.1 ± 4.5 days and 24.8 ± 14 days for the sarcopenic group, respectively. Overall, in-hospital mortality was 5.9% (8/135). The 30-day, 1-year, 3-year and 5-year mortality was 10.4% (14/135), 20% (27/135), 28.1% (38/135) and 31.1% (42/135), respectively. Eleven patients (8.1%) were lost during follow-up with no further contact possible. Long-term mortality according to SMI and TPMI is shown in Figure 4 and Figure 5, respectively. There was no difference in the long-term mortality rates between sarcopenic and non-sarcopenic patients regardless of the stratification method used (*p* = 0.454 for SMI and *p* = 0.209 for TPMI). According to SMI, 30-day mortality of sarcopenic patients was significantly lower in comparison to non-sarcopenic patients (1/45, 2.2% vs. 13/90, 14.4%, *p* = 0.028). There was no difference in the 1-year and 3-year mortality rates (*p* = 0.576 and *p* = 0.507 for 1-year and 3-year follow-up, respectively). There was no difference in the 30-day, 1-year and 3-year mortality rates between sarcopenic and non-sarcopenic patients after TPMI stratification (*p* = 0.324, *p* = 0.977 and *p* = 0.309 for 30-day, 1-year and 3-year follow-up, respectively).

A multivariate regression analysis demonstrated that age was the only significant predictor of shorter survival for both measurement methods (hazard ratio 1.07, 95% confidence interval 1.02–1.12, *p* = 0.003 for SMI and hazard ratio 1.08, 95% confidence interval 1.02–1.12, *p* = 0.003 for TPMI) after adjustments were made for age, body mass index, coronary artery disease, chronic obstructive pulmonary disease, diabetes, smoking and chronic kidney disease (Table 3).

### 3.3. Secondary Endpoints

Postoperative complications are shown in Table 4. Eleven patients had major adverse cardiac events, twenty-two patients developed pulmonary complications and fourteen patients developed acute kidney injury postoperatively, defined as a >50% decrease of glomerular filtration rate, with ten of them requiring temporary hemodialysis. According to TPMI, patients were at higher risk for development of pulmonary complications postoperatively (*p* = 0.03).

**Figure 6:**
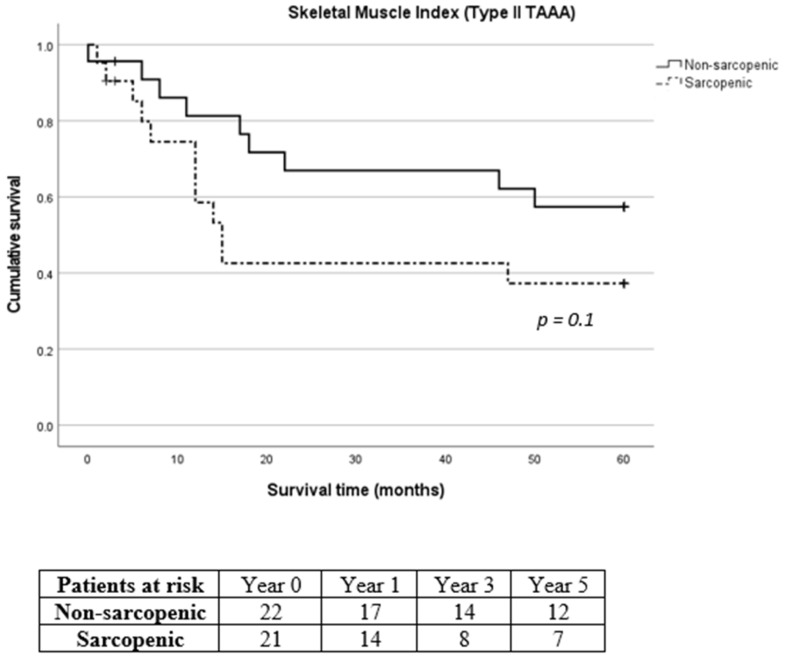
Long-term survival of sarcopenic and non-sarcopenic patients with type II thoracoabdominal aortic aneurysm after stratification using skeletal muscle index. The Kaplan–Meier curves were compared using the log-rank test.

**Figure 7:**
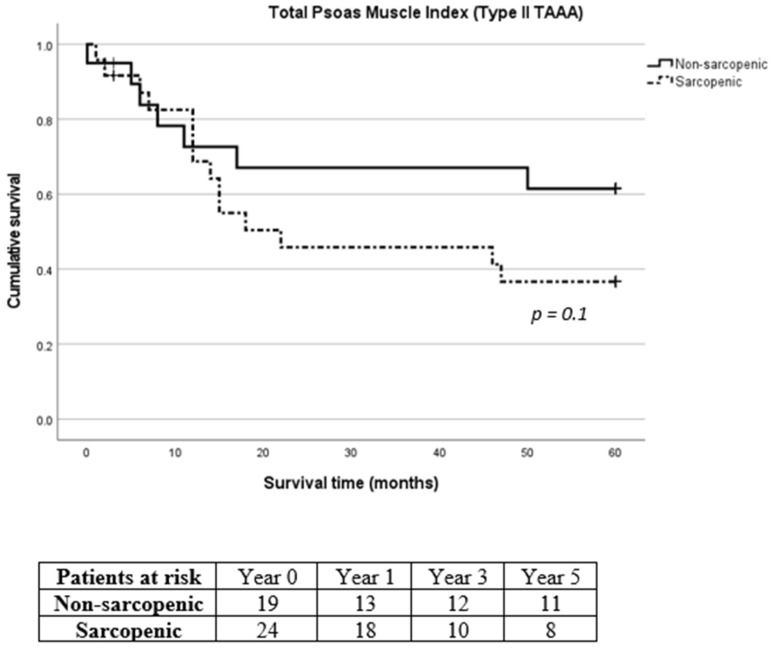
Long-term survival of sarcopenic and non-sarcopenic patients with type II thoracoabdominal aortic aneurysm after stratification using total psoas muscle index. The Kaplan–Meier curves were compared using the log-rank test.

Due to the considerable prevalence of patients with reduced muscle mass and type II TAAA, we exclusively analyzed the survival rates of patients diagnosed specifically with type II TAAA. No difference was recorded in 30-day, 1-year, 3-year and 5-year mortality between sarcopenic and non-sarcopenic patients with type II TAAA (*p* = 0.961, *p* = 0.134, *p* = 0.098 and *p* = 0.136 for 30-day, 1-year, 3-year and 5-year mortality for SMI and *p* = 0.883, *p* = 0.865, *p* = 0.280 and *p* = 0.188 for 30-day, 1-year, 3-year and 5-year mortality for TPMI, respectively, Figure 6 and Figure 7, respectively).

## 4. Discussion

The role of sarcopenia as a prognostic tool in vascular surgery patients has been the subject of several studies in recent years [17]. The hypothesis that reduced muscle mass is related to higher mortality and reduced outcomes in patients following open or endovascular repair of aortic aneurysms has been studied by many authors, with the results being controversial [9,11,18,19]. One of the issues appears to be the presence of various methods for measuring muscle mass and the cutoff values used to classify patients as sarcopenic and non-sarcopenic. Most commonly used method is the measurement of either total skeletal mass area or total psoas mass area on the level of third lumbar vertebrae and then normalization of the values according to the patients’ height [15,16]. In a recent study using SMI for patient stratification, Ghaffarian et al. compared the long-term survival between sarcopenic and non-sarcopenic patients after operative and non-operative management of thoracoabdominal aortic aneurysms. His results demonstrated a better long-term survival for non-sarcopenic patients regardless of management [13]. In another report from Chatterjee et al. involving 392 patients stratified according to TPMI, decreased muscle mass was not associated with higher early mortality in patients undergoing surgical management of thoracoabdominal aortic aneurysms. While the findings of the study indicated a poorer midterm survival, this was not independently linked to sarcopenia [12]. Finally, in a systematic review and meta-analysis from Nana et al. lower muscle mass did not seem to impact early mortality in patients undergoing treatment for thoracoabdominal aortic aneurysms [20]. In these studies, either skeletal mass index or psoas muscle index was used for patient stratification.

In our study, we wanted to test the validity of both measurement methods and their impact in survival and perioperative outcomes. Our data showed no difference in the long-term mortality of sarcopenic and non-sarcopenic patients for both SMI and TPMI. Additionally, regardless of the stratification method used, there was a significant association between patients with lower muscle mass and type II thoracoabdominal aortic aneurysm. Other significant results in our study were the longer hospital stay of sarcopenic patients according to SMI and the development of pulmonal complications after TPMI stratification. An association between longer hospital stay and lower muscle mass has been already reported, such as in the works of Thurston et al. and Newton et al. [21,22]. Concerning pulmonary complications, the authors think that despite the validity of our data, current evidence to establish a link between pulmonary complications and reduced muscle mass is very weak, as this significance was observed solely through one stratification method.

It appears that patient stratification according to image-based measurements alone cannot reliably be used as a prognostic tool in patients undergoing management for thoracoabdominal aortic aneurysms. Additionally, the arbitrarily selected threshold at the lower tertile appears inadequate for accurately categorizing patients into sarcopenic and non-sarcopenic groups. In the report from Chatterjee et al. which used TPMI for patient stratification, the cutoff values for men and women were 9.1 cm^2^/m^2^ and 6.2 cm^2^/m^2^, respectively [12]. According to these values, 94.8% of our patients would have been classified as sarcopenic (128/135). In a consensus statement of the European Association for Cardio-Thoracic Surgery and the European Association of Preventive Cardiology of the European Society of Cardiology cutoff values of 8.5 cm^2^/m^2^ for men and 6.5 cm^2^/m^2^ for women regarding psoas muscle index were proposed [23]. The European Working Group on Sarcopenia in Older People (EWGSOP) in its recent guidelines proposed Find cases–Assess–Confirm–Severity (FACS), an algorithm used primarily in identifying patients at risk for sarcopenia and then assessing and confirming the diagnosis. This algorithm utilizes various tools such as SARC-F, a questionnaire used to screen patients for self-reported signs suggestive of sarcopenia, grip strength, chair stand test, skeletal muscle mass, gait speed and timed up-and-go test [24]. According to the EWGSOP, muscle strength plays an important role in assessment of sarcopenia, since it is recognized as a better predictor than muscle mass in regard to adverse outcomes [24,25,26]. The authors believe that future studies investigating the role of muscle mass in vascular surgery patients should be of a prospective nature and utilize the aforementioned algorithm in order to better stratify patients as sarcopenic and non-sarcopenic and produce safer results.

The main limitations of our study are its retrospective nature and the assessment of the CT images by only one observer. Many studies employ a primary and a secondary blinded investigator for image assessment in order to avoid measurement errors. In our study, we deliberately chose only one trained radiologist and opted for Horos (a free, open source medical image viewer) for image assessment to examine the feasibility of obtaining valid results quickly and from vascular surgeons rather than trained radiologists.

## 5. Conclusions

Our study showed no effect of sarcopenia on long-term survival of patients undergoing complex endovascular aortic management for thoracoabdominal aortic aneurysms after image-based stratification using SMI and TPMI. Patient stratification according to CT image-based measurements alone and arbitrarily definition of sarcopenia seem to be a weak prognostic tool. More studies of a prospective nature utilizing the algorithm of the EWGSOP are required in order to better assess the role of muscle mass in vascular surgery patients.

## Figures and Tables

**Table 1:** Patient demographics and health characteristics.

Variable	Total No. (%)
Male/Female ratio	86 (63.7%)/49 (36.3%)
Mean patient age (y.o ± SD)	69.7 ± 8.6
BMI (kg/m^2^ ± SD)	26.9 ± 4.6
ASA grading	
- ASA II	17 (12.6%)
- ASA III	104 (77%)
- ASA IV	14 (10.4%)
Type of TAAA	
- Type I	8 (5.9%)
- Type II	44 (32.6%)
- Type III	47 (34.8%)
- Type IV	30 (22.2%)
- Type V	6 (4.4%)
Prior vascular surgery	60 (44.4%)
- Open repair	44 (32.6%)
- Endovascular	20(14.8%)
- Both	5 (3.6%)
CAD	59 (43.7%)
Hypertension	129 (95.6%)
Stroke	25 (18.5%)
COPD	43 (31.9%)
Diabetes	14 (10.4%)
Smoking	56 (41.5%)
CKD	53 (39.3%)
- GFR < 60 (mL/min/1.73 qm)	40 (29.6%)
- GFR < 30 (mL/min/1.73 qm)	13 (9.6%)

No. = number; y.o = years old; SD = standard deviation; BMI = body mass index; ASA = American Society of Anesthesiology score; TAAA = thoracoabdominal aortic aneurysm according to Crawford classification; CAD = coronary artery disease; Hypertension = blood pressure > 130/80 mmHg; COPD = chronic obstructive pulmonary disease: Diabetes = HbA1C > 7%; Hypercholesterolemia = total cholesterol levels > 200 mg/dL; CKD = chronic kidney disease; GFR = glomerular filtration rate.

**Table 2:** Patient characteristics according to muscle measurement method.

Variable	Skeletal Muscle Index (SMI)			Total Psoas Muscle Index (TPMI)		
	Sarcopenic (*n* = 45)	Non Sarcopenic (*n* = 90)	*p*-Value	Sarcopenic (*n* = 45)	Non Sarcopenic (*n* = 90)	*p*-Value
Male/Female ratio	29 (64.4%)/16 (35.6%)	57 (63.3%)/33 (36.7%)	*0.89 ^a^*	29 (64.4%)/16 (35.6%)	57 (63.3%)/33 (36.7%)	*0.89 ^a^*
Mean patient age (y.o ± SD)	70.9 ± 8.5	69.1 ± 8.7	*0.3 ^b^*	70.6 ± 10.2	69.2 ± 7.7	*0.15 ^b^*
BMI (kg/m^2^ ± SD)	24.7 ± 4.2	28.1 ± 4.4	*0.5 ^b^*	24.9 ± 4.2	28 ± 4.4	*0.39 ^b^*
ASA grading			*0.92 ^a^*			*0.34 ^a^*
- ASA II	5 (11.1%)	12 (13.3%)		3 (6.7%)	14 (15.6%)	
- ASA III	35 (77.8%)	69 (76.7%)		37 (82.2%)	67 (74.4%)	
- ASA IV	5 (11.1%)	9 (10%)		5 (11.1%)	9 (10%)	
Type of TAAA						
- Type I	1 (2.2%)	7 (7.8%)	*0.19 ^c^*	**0 (0%)**	**8 (8.9%)**	***0.03** ^c^*
- Type II	**21 (46.7%)**	**23 (25.6%)**	***0.01** ^a^*	**24 (53.3%)**	**20 (22.2%)**	***<0.01*** *^a^*
- Type III	14 (31.1%)	33 (36.7%)	*0.52 ^a^*	13 (28.9%)	34 (37.8%)	*0.3 ^a^*
- Type IV	8 (17.8%)	22 (24.4%)	*0.38 ^a^*	7 (15.6%)	23 (25.6%)	*0.18 ^a^*
- Type V	1 (2.2%)	5 (5.6%)	*0.37 ^c^*	1 (2.2%)	5 (5.6%)	*0.37 ^c^*
Prior vascular surgery	24 (53.3%)	36 (40%)	*0.14 ^a^*	25 (55.6%)	35 (38.9%)	*0.66 ^a^*
- Open repair	18 (40%)	26 (28.9%)	*0.19 ^a^*	19 (42.2%)	25 (27.8%)	*0.91 ^a^*
- Endovascular	8 (17.8%)	12 (13.3%)	*0.49 ^a^*	6 (13.3%)	14 (15.6%)	*0.73 ^a^*
- Both	1 (2.2%)	4 (4.4%)	*0.51*	1 (2.2%)	4 (4.4%)	*0.51*
CAD	20 (44.4%)	39 (43.3%)	*0.9*	21 (46.7%)	38 (42.2%)	*0.62*
Hypertension	44 (97.8%)	85 (94.4%)	*0.37*	44 (97.8%)	85 (94.4%)	*0.37*
Stroke	11 (24.4%)	14 (15.6%)	*0.21*	11 (24.4%)	14 (15.6%)	*0.21*
COPD	14 (31.1%)	29 (32.2%)	*0.89*	13 (28.9%)	30 (33.3%)	*0.6*
Diabetes	6 (13.3%)	8 (8.9%)	*0.42*	4 (8.9%)	10 (11.1%)	*0.69*
Smoking	15 (33.3%)	41 (45.6%)	*0.17*	15 (33.3%)	41 (45.6%)	*0.17*
CKD	18 (40%)	35 (38.9%)	*0.9*	17 (37.8%)	36 (40%)	*0.8*
- GFR < 60 (ml/min/1.73 qm)	15 (33.3%)	25 (27.8%)		14 (31.1%)	26 (28.9%)	
- GFR < 30 (ml/min/1.73 qm)	3 (6.7%)	10 (11.1%)		3 (6.7%)	10 (11.1%)	

y.o = years old; SD = standard deviation; BMI = body mass index; ASA = American Society of Anesthesiology score; TAAA = thoracoabdominal aortic aneurysm according to Crawford classification; CAD = coronary artery disease; Hypertension = blood pressure > 130/80 mmHg; COPD = chronic obstructive pulmonary disease: Diabetes = HbA1C > 7%; Hypercholesterolemia = total cholesterol levels > 200 mg/dL; CKD = chronic kidney disease; GFR = glomerular filtration rate. *^a^* = Pearson chi-square test; *^b^* = Whitney U test; *^c^* = Fisher’s exact test.

**Table 3:** Multivariate Cox regression model of survival among patients stratified using skeletal muscle index and total psoas muscle index.

	Skeletal Muscle Index HR 1.77, 95% CI 0.86–3.6, *p = 0.11*			Total Psoas Muscle Index HR 0.82, 95% CI 0.43–1.56, *p = 0.55*		
Variable	HR	95% CI	*p*-Value	HR	95% CI	*p*-Value
Age	1.07	1.02–1.12	***0.003***	1.08	1.02–1.12	***0.003***
Body mass index	0.93	0.86–1	*0.07*	0.95	0.89–1.02	*0.2*
CAD	0.73	0.37–1.43	*0.36*	0.7	0.36–1.34	*0.28*
COPD	0.62	0.31–1.24	*0.18*	0.63	0.32–1.25	*0.18*
Diabetes	0.95	0.32–2.84	*0.93*	1.03	0.35–3.07	*0.94*
Smoking	0.94	0.48–1.83	*0.86*	0.87	0.45–1.71	*0.7*
CKD	1.71	0.86–3.41	*0.12*	1.71	0.86–3.38	*0.12*

HR = hazard ration; CI = confidence interval; CAD = coronary artery disease; COPD = chronic obstructive pulmonary disease; CKD = chronic kidney disease.

**Table 4:** Total postoperative complications and complications according to measurement stratification.

		Skeletal Muscle Index			Total Psoas Muscle Index		
Postoperative Complications	Total No. (%)	Sarcopenic (*n* = 45)	Non-Sarcopenic (*n* = 90)	*p* Value	Sarcopenic (*n* = 45)	Non-Sarcopenic (*n* = 90)	*p* Value
MACE	13 (9.6%)	7 (15.6%)	6 (6.7%)	*0.09 ^a^*	6 (13.3%)	7 (7.8%)	*0.3 ^a^*
Pulmonal	28 (20.7%)	12 (26.7%)	16 (17.8%)	*0.23 ^a^*	**14 (31.3%)**	**14 (15.6%)**	***0.03*** *^a^*
AKI	21 (15.6%)	7 (15.6%)	14 (15.6%)	*1 ^a^*	9 (20%)	12 (13.3%)	*0.45 ^a^*
Hemodialysis	10 (7.4%)	4 (8.9%)	6 (6.7%)	*0.64 ^b^*	5 (11.1%)	5 (5.6%)	*0.24 ^b^*

No. = number; MACE = major adverse cardiac event; AKI = acute kidney injury, decrease in glomerular filtration rate > 50% postoperatively. *^a^* = Pearson chi-square test; *^b^* = Fisher’s exact test.

## Data Availability

The data presented in this study are available on request from the corresponding author. The data are not publicly available due to ethical and privacy compliance.
